# Bis[μ-3-(pyridin-2-yl)pyrazolato]bis­[acetato­(3,5-dimethyl-1*H*-pyrazole)­nickel(II)]

**DOI:** 10.1107/S2414314624008101

**Published:** 2024-08-30

**Authors:** Thangamuniyandi Pilavadi, Soundararajan Krishnan, Nagarajan Loganathan

**Affiliations:** a School of Chemistry Bharathidasan University, Tiruchirappalli 620 024 Tamilnadu India; b Department of Chemistry, Periyar Maniammai Institute of Science and Technology, Vallam-613403, Thanjavur, Tamil Nadu, India; Purdue University, USA

**Keywords:** coordination compound, nickel, 3,5-di­methyl­pyrazole, 3-(pyridin-2-yl) pyrazole, heteroleptic complex, crystal structure

## Abstract

The title compound, [Ni_2_(C_8_H_6_N_3_)_2_(C_2_H_3_O_2_)_2_(C_5_H_8_N_2_)_2_] or [Ni(*μ*-OOCCH_3_)(2-PyPz)(Me_2_PzH)]_2_ (**1**) [2-PyPz = 3-(pyridin-2-yl) pyrazole; Me_2_PzH = 3,5-dimethyl pyrazole] was synthesized from Ni(OOCCH_3_)_2_·4H_2_O, 2-PyPzH, Me_2_PzH and tri­ethyl­amine as a base. Compound **1** {[Ni_2_(C_30_H_34_N_10_Ni_2_O_4_)]} at 100 K has monoclinic (*P*2_1_/*n*) symmetry and the mol­ecules have crystallographic inversion symmetry. Mol­ecules of **1** comprise an almost planar dinuclear Ni^II^ core with an N_4_O_2_ coordination environment. The equatorial plane consists of N_3_,O coordination derived from one of the bidentate acetate O atoms and three of the N atoms of the chelating 2-PyPz ligand while the axial positions are occupied by neutral Me_2_PzH and the second O atom of the acetate unit. The Ni atoms are bridged by the nitro­gen atom of a deprotonated 2-PyPz ligand. Compound **1** exhibits various inter- and intra­molecular C—H⋯O and N—H⋯O hydrogen bonds.

## Abstract 2

The title compound is a dimeric nickel(II) coordination compound containing two different substituted pyrazoles ligands, namely 3,5-di­methyl­pyrazole and 3-(pyridin-2-yl) pyrazole along with acetate.

## Graphical abstract

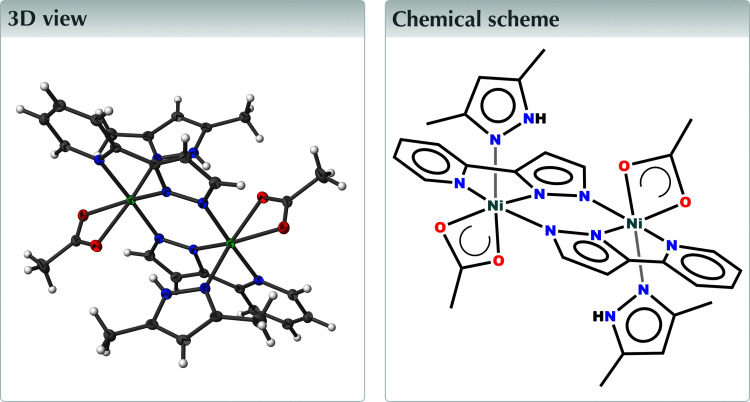

## Structure description

Noble metals such as palladium, platinum or iridium are widely used in catalysis due to their desirable properties such as the ability to tolerate variable coordination states and oxidation states that predispose them towards catalysing two-electron redox processes, while at the same time also being sufficiently stable and thermally stable to be of practical use. A major drawback is, however, their high price and limited availability. As an alternative to scarce 4 and 5*d* metals, their more earth-abundant 3*d* congeners have been investigated, and in particular several nickel-catalysed organic transform­ation strategies were developed and established (Wilke, 1988 ▸; Keim, 1990 ▸; Montgomery, 2004 ▸; Tasker *et al.*, 2014 ▸; Diccianni *et al.*, 2020 ▸). These include C—C and C—*X* (*X* = heteroatom) cross-coupling (Rosen *et al.*, 2011 ▸), cyclo­addition (Lautens *et al.*, 1996 ▸; Komagawa *et al.*, 2013 ▸), asymmetric hydrogenation (Vermaak *et al.*, 2024 ▸), photo-redox catalysis (Milligan *et al.*, 2019 ▸; Cuesta-Galisteo *et al.*, 2024 ▸), reductive coupling (Day *et al.*, 2023 ▸) and reductive cyclization reactions (Montgomery, 2004 ▸) to name just a few. The inability of nickel to catalyse two-electron transformations can be overcome by the placement of more than one metal atom at the catalytic centre, and dinuclear nickel complexes show an enhanced catalytic activity and a higher robustness that can be traced back to the synergistic inter­action between the two metals in the active site (Uyeda & Farley 2021 ▸; Xu *et al.*, 2020 ▸). Nickel is also a micronutrient and essential for the biosynthesis of hydrogenase, carbon monoxide de­hydrogenase (CODH) and urease. These enzymes require more than one metal active site to catalyse the enzymatic process. This also substanti­ates the crucial role of the presence of more than one metal centre for 3*d*-metal-based catalysts.

**Figure 1:**
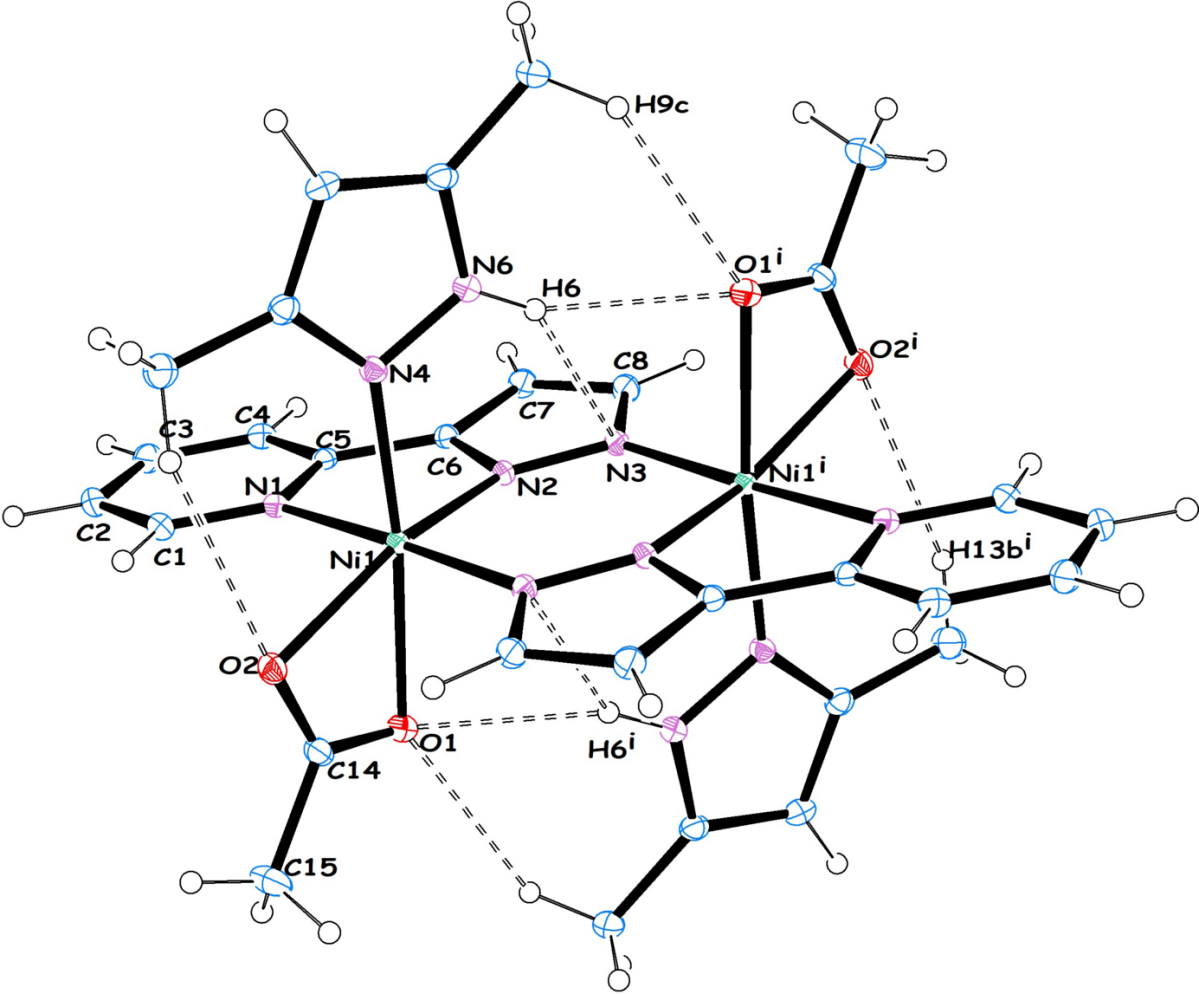
The mol­ecule of **1** (with 50% displacement ellipsoids) with the unlabelled atoms related by crystallographic inversion symmetry (−*x*, −*y*, 1 − *z*). Intra­molecular C—H⋯O, N—H⋯O and N—H⋯N hydrogen bonds are shown as dashed lines.

We are inter­ested in synthesizing dimeric Ni^II^ complexes utilizing chelating ligands such as 2-PyPzH [3-(2-pyridyl)pyrazole, C_8_H_7_N_3_]. The use of pyrazole ligands in coordination and organometallic chemistry is well established (Trofimenko, 1972 ▸; Mukherjee, 2000 ▸; Halcrow, 2009 ▸; Viciano-Chumillas *et al.*, 2010 ▸). 2-PyPzH usually forms planar dimeric [*M*(*μ*-2-PyPz)_2_]_2_ units that are thermally stable. Copper-based dimeric complexes with a {[Cu(*μ*-2-PyPz)_2_]_2_}_*n*_ core have been described (Jeffery *et al.*, 1997 ▸; Hu *et al.*, 2006 ▸; Das *et al.*, 2019 ▸). However, to the best of our knowledge, the analogous nickel complex with an [Ni(*μ*-2-PyPz)_2_]_*n*_ core is unknown. Thus, a reaction was carried out between nickel(II)acetate tetra­hydrate, 2-PyPzH as the primary ligand and highly lipophilic 3,5-di­methyl­pyrazole (Me_2_PzH) as an ancillary ligand and a small excess of tri­ethyl­amine base in methanol solvent. This was done in a 1:1:5:3.5 ratio, which resulted in the formation of a green solid, which was then recrystallized from methanol solvent to obtain blue crystals of [Ni_2_(*μ*-OOCCH_3_)_2_(2-PyPz)_2_(Me_2_PzH)_2_] (**1**). Inter­estingly, the initial reaction between nickel(II)acetate tetra­hydrate, 2-PyPzH and tri­ethyl­amine base in a 1:1:1.5 stoichiometry failed and led to an intra­ctable mixture. However, the addition of a large excess of Me_2_PzH allowed us to isolate the soluble mol­ecular assembly of **1** (Fig. 1 ▸).

Compound **1** crystallizes in the monoclinic *P*2_1_/*n* space group, in which the asymmetric unit contains half of the mol­ecule. Compound **1** is a dinuclear heteroleptic nickel(II) complex consisting of two each of anionic 2-PyPz, anionic CH_3_COO^−^ and neutral Me_2_PzH ligands and the complex mol­ecules have crystallographic inversion symmetry. Overall, the two nickel atoms (Ni1 and Ni1^i^) are bridged through the 2-PyPz ligand and each Ni atom has an N_4_O_2_ octa­hedral coordination environment around it. The three N-donors (N1, N2 and N3^i^) are derived from the 2-PyPz unit, which forms the basal plane of the dimer while the fourth N-coordination (N4) is obtained from the axial neutral Me_2_PzH ligand. The acetate ligand (O1 and O2) exhibits a *syn*–*syn* symmetric binding mode (κ^2^ mode) in which O2 is in the equatorial position while the sixth axial position is occupied by O1.

The following is a summary of the bonding parameters found in compound **1** in which each Ni atom exhibits three different Ni—N distances and two different Ni—O distances. The Ni—N distance involving the anionic pyrazole unit is shorter [Ni1—N2 = 2.0245 (12); Ni1—N3^i^ = 2.0409 (13) Å] compared to the pyridinic N of 2-PyPz [Ni1—N1 = 2.0964 (13) Å] and the neutral Me_2_PzH ligand [Ni1—N4 = 2.0884 (12) Å]. Additionally, the axial Ni—O distances are longer [Ni1—O1 = 2.1848 (11) Å] than the equatorial distance [Ni1— O2 = 2.1232 (11) Å]. Furthermore, the C—O distances are not equal [C14—O1 = 1.2576 (19); C14—O2 = 1.2641 (19) Å]. It is noteworthy that the dimeric [Ni(*μ*-2-PyPz)(COOCH_3_)]_2_ unit is almost planar, with the two basal *trans* angles being less than 180° [O1—Ni1—N4 = 170.18 (5); N1—Ni1—N3^i^ = 177.68 (5)°]. The angle between the two apical positions is the most acute [O2—Ni1—N2 = 157.80 (5)°]. Finally, of the twelve right angles around Ni1, seven are closer to 90° [average O—Ni—N = 89.16 (4) and average N—Ni—N = 91.03 (6)°], and the remaining three are obtuse [N2—Ni1—N3^i^ = 100.88 (5); O1—Ni1—N2 = 99.02 (5); O2—Ni1—N4 = 109.07 (5)°].

**Figure 2:**
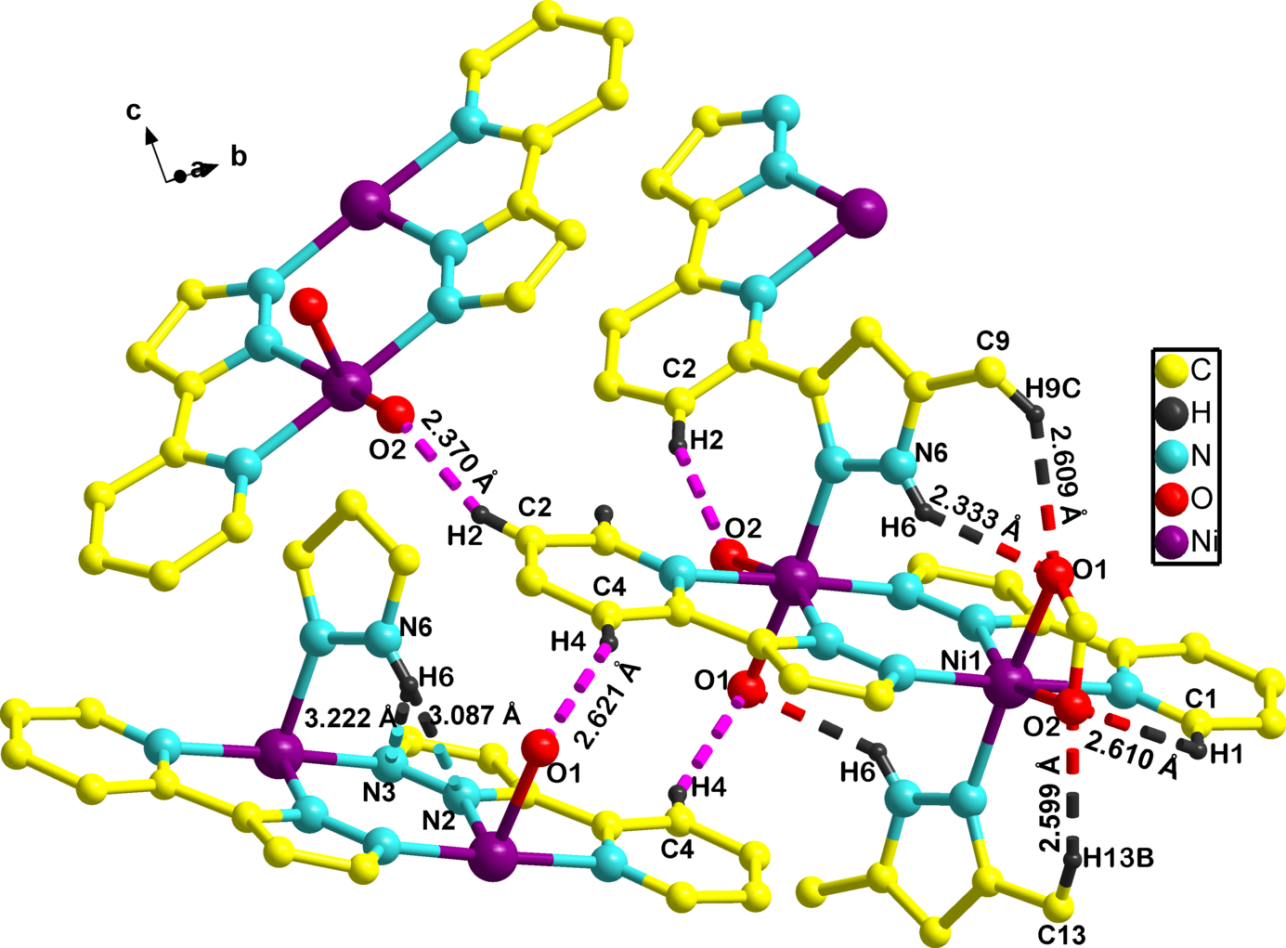
Perspective view of **1** showing the intra- (red and black dotted lines) and inter­molecular C—H⋯O (pink dotted lines) and intra­molecular N—H⋯N (blue and black dotted lines) inter­actions with bond distances (several atoms were removed for clarity).

Compound **1** exhibits several intra- and inter­molecular hydrogen bonds (Table 1 ▸, Fig. 2 ▸), with atom N6 of Me_2_PzH forming intra­molecular hydrogen bonds with O1 of the acetate (N6—H6⋯O1^i^ and the reciprocal N6^i^—H6^i^⋯O1 3.0800 (17) Å; symmetry code: (i) −*x*, −*y*, −*z* + 1), with N2 [N6—H6⋯N2 2.9931 (18) Å] and N3 [N6—H6⋯N3 3.3065 (18) Å] of 2-PyPz, while the two O atoms of acetate (O1 and O2) inter­act with the pyridine C—H of 2-PyPz and pyrazolyl C—H of Me_2_PzH. Thus, the hydrogen bonding between C2—H2⋯O2^ii^ [3.2692 (19) Å; symmetry code: (ii) −*x* + 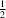, *y* + 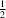, −*z* + 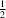] and C4—H4⋯O1^iii^ [3.4696 (19) Å; symmetry code: (iii) −*x*, −*y* + 1, −*z* + 1] are inter­molecular in nature while the C9—H9*C*⋯O1^i^ [3.426 (2) Å], C1—H1⋯O2 [3.1281 (19) Å] and C13—H13*B*⋯O2 [3.530 (2) Å] are of intra­molecular type.

## Synthesis and crystallization

0.5 mmol of Ni(OOCCH_3_)_2_·4H_2_O (0.1244 g) was dissolved in 30 ml of methanol. Then, 0.5 mmol of 2-PyPzH (0.0726 g) and 0.79 mmol of tri­ethyl­amine (0.11 ml) were added to the solution. Upon addition of these, the solution became milky white and insoluble. It was stirred for 2 h. After every 30 minutes of stirring, 0.5 mmol of lipophilic Me_2_PzH (0.2402 g, 2.5 mmol) and equal portions of tri­ethyl­amine (0.11 ml, 0.79 mmol) were added. The solution slowly turned green and was further stirred for 12 h. It was then filtered and solvents were evaporated *in vacuo* to obtain a pale-green solid. Finally, the solid was recrystallized from methanol solution, which afforded blue crystals of **1**. Crystal yield 45% [based on Ni(OOCCH_3_)_2_·4H_2_O], m.p. 212°C. ESI–MS: [*M* − 2H]^+^ 713.479; [*M*_1_ + Li]^+^ where [*M*_1_ = *M*-2(Me_2_PzH)-CH_3_CO] 487.309. FT–IR (KBr, ν, cm^−1^): 3122 (*s*), 3114 (*s*), 3000 (*m*), 2937 (*s*), 2738 (*m*), 2677 (*s*), 2015 (m, br), 1470 (*m*), 1307 (*m*), 1268 (*m*), 1407 (*m*), 1094 (*s*, *br*), 1032 (*m*), 941 (*s*), 898 (*s*), 855 (*m*), 811 (*s*), 624 (*s*), 554 (*m*).

## Refinement

Crystal data, data collection and structure refinement details are summarized in Table 2 ▸.

## Supplementary Material

Crystal structure: contains datablock(s) I. DOI: 10.1107/S2414314624008101/zl4073sup1.cif

Structure factors: contains datablock(s) I. DOI: 10.1107/S2414314624008101/zl4073Isup2.hkl

CCDC reference: 2346359

Additional supporting information:  crystallographic information; 3D view; checkCIF report

## Figures and Tables

**Table 1:** Hydrogen-bond geometry (Å, °)

*D*—H⋯*A*	*D*—H	H⋯*A*	*D*⋯*A*	*D*—H⋯*A*
N6—H6⋯N2	0.859 (18)	2.571 (18)	2.9931 (18)	111.4 (14)
N6—H6⋯N3	0.859 (18)	2.591 (19)	3.3065 (18)	141.4 (16)
N6—H6⋯O1^i^	0.859 (18)	2.332 (19)	3.0800 (17)	145.6 (16)
C1—H1⋯O2	0.95	2.61	3.1281 (19)	115
C2—H2⋯O2^ii^	0.95	2.37	3.2692 (19)	158
C4—H4⋯O1^iii^	0.95	2.62	3.4696 (19)	149
C9—H9*C*⋯O1^i^	0.98	2.61	3.426 (2)	141
C13—H13*B*⋯O2	0.98	2.60	3.530 (2)	159

Symmetry codes: (i) 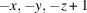; (ii) 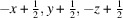; (iii) 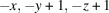.

**Table 2:** Experimental details

Crystal data	
Chemical formula	[Ni_2_(C_8_H_6_N_3_)_2_(C_2_H_3_O_2_)_2_(C_5_H_8_N_2_)_2_]
*M*_r_	716.09
Crystal system, space group	Monoclinic, *P*2_1_/*n*
Temperature (K)	100
*a*, *b*, *c* (Å)	11.1045 (7), 9.1489 (6), 15.8088 (11)
β (°)	92.210 (1)
*V* (Å^3^)	1604.88 (18)
*Z*	2
Radiation type	Mo *K*α
μ (mm^−1^)	1.23
Crystal size (mm)	0.12 × 0.10 × 0.10

Data collection	
Diffractometer	Bruker *APEX*
Absorption correction	Multi-scan (*SADABS*; Krause *et al.*, 2015 ▸
*T*_min_, *T*_max_	0.875, 0.905
No. of measured, independent and observed [*I* > 2σ(*I*)] reflections	10468, 3946, 3617
*R*_int_	0.025
(sin θ/λ)_max_ (Å^−1^)	0.667

Refinement	
*R*[*F*^2^ > 2σ(*F*^2^)], *wR*(*F*^2^), *S*	0.030, 0.079, 1.04
No. of reflections	3946
No. of parameters	214
H-atom treatment	H atoms treated by a mixture of independent and constrained refinement
Δρ_max_, Δρ_min_ (e Å^−3^)	0.44, −0.27

Computer programs: *SMART* and *SAINT* (Bruker, 2012 ▸), *SHELXT* (Sheldrick, 2015*a* ▸), *SHELXL2019/2* (Sheldrick, 2015*b* ▸), *ORTEP-3 for Windows* (Farrugia, 2012 ▸), *DIAMOND* (Brandenburg *et al.*, 2014 ▸) and *publCIF* (Westrip, 2010 ▸).

## full crystallographic data

### Bis[µ-3-(pyridin-2-yl)pyrazolato]bis[acetato(3,5-dimethyl-1H-pyrazole)nickel(II)] Crystal data

**Table:**

[Ni_2_(C_8_H_6_N_3_)_2_(C_2_H_3_O_2_)_2_(C_5_H_8_N_2_)_2_]	*F*(000) = 744
*M**_r_* = 716.09	*D*_x_ = 1.482 Mg m^−^^3^
Monoclinic, *P*2_1_/*n*	Mo *K*α radiation, λ = 0.71073 Å
*a* = 11.1045 (7) Å	Cell parameters from 5987 reflections
*b* = 9.1489 (6) Å	θ = 2.6–28.3°
*c* = 15.8088 (11) Å	µ = 1.23 mm^−^^1^
β = 92.210 (1)°	*T* = 100 K
*V* = 1604.88 (18) Å^3^	Prism, blue
*Z* = 2	0.12 × 0.10 × 0.10 mm

### Bis[µ-3-(pyridin-2-yl)pyrazolato]bis[acetato(3,5-dimethyl-1H-pyrazole)nickel(II)] Data collection

**Table:**

Bruker APEX diffractometer	*R*_int_ = 0.025
Radiation source: sealed tube	θ_max_ = 28.3°, θ_min_ = 2.2°
φ and ω scans	*h* = −14→14
Absorption correction: multi-scan (SADABS; Krause *et al.*, 2015	*k* = −12→12
*T*_min_ = 0.875, *T*_max_ = 0.905	*l* = −21→14
10468 measured reflections	4 standard reflections every 22 reflections
3946 independent reflections	intensity decay: none
3617 reflections with *I* > 2σ(*I*)	

### Bis[µ-3-(pyridin-2-yl)pyrazolato]bis[acetato(3,5-dimethyl-1H-pyrazole)nickel(II)] Refinement

**Table:**

Refinement on *F*^2^	Primary atom site location: difference Fourier map
Least-squares matrix: full	Secondary atom site location: inferred from neighbouring sites
*R*[*F*^2^ > 2σ(*F*^2^)] = 0.030	Hydrogen site location: mixed
*wR*(*F*^2^) = 0.079	H atoms treated by a mixture of independent and constrained refinement
*S* = 1.04	*w* = 1/[σ^2^(*F*_o_^2^) + (0.0457*P*)^2^ + 0.5264*P*] where *P* = (*F*_o_^2^ + 2*F*_c_^2^)/3
3946 reflections	(Δ/σ)_max_ < 0.001
214 parameters	Δρ_max_ = 0.44 e Å^−^^3^
0 restraints	Δρ_min_ = −0.27 e Å^−^^3^

### Bis[µ-3-(pyridin-2-yl)pyrazolato]bis[acetato(3,5-dimethyl-1H-pyrazole)nickel(II)] Special details

**Table:**

Geometry. All esds (except the esd in the dihedral angle between two l.s. planes) are estimated using the full covariance matrix. The cell esds are taken into account individually in the estimation of esds in distances, angles and torsion angles; correlations between esds in cell parameters are only used when they are defined by crystal symmetry. An approximate (isotropic) treatment of cell esds is used for estimating esds involving l.s. planes.
Refinement. All the non-hydrogen atoms were refined anisotropically using full-matrix least-square procedures while carbon bound hydrogen atoms were included in idealized positions and the methyl CH_3_ were allowed to rotate using a riding model. C—H bonds were constrained to 0.95 Å for aromatic C—H (*U*_iso_(H) = 1.2 *U*_eq_(C)) and 0.98 Å for CH_3_ [*U*_iso_(H) = 1.5 *U*_eq_(C)] units, respectively. The N—H proton was added from the difference Fourier map and refined with *U*_iso_(H) = 1.2 *U*_eq_(N).

### Bis[µ-3-(pyridin-2-yl)pyrazolato]bis[acetato(3,5-dimethyl-1H-pyrazole)nickel(II)] Fractional atomic coordinates and isotropic or equivalent isotropic displacement parameters (Å^2^)

**Table:**

	*x*	*y*	*z*	*U*_iso_*/*U*_eq_	
C1	0.11107 (14)	0.40306 (17)	0.31691 (10)	0.0189 (3)	
H1	0.177134	0.357829	0.290855	0.023*	
C2	0.08185 (14)	0.54516 (18)	0.29476 (10)	0.0212 (3)	
H2	0.125431	0.595694	0.253272	0.025*	
C3	−0.01268 (15)	0.61225 (16)	0.33456 (11)	0.0213 (3)	
H3	−0.033162	0.710881	0.321898	0.026*	
C4	−0.07701 (13)	0.53426 (17)	0.39297 (9)	0.0184 (3)	
H4	−0.142463	0.578183	0.420430	0.022*	
C5	−0.04392 (13)	0.39056 (15)	0.41058 (9)	0.0152 (3)	
C6	−0.10578 (12)	0.29590 (16)	0.46928 (9)	0.0154 (3)	
C7	−0.21072 (13)	0.31154 (17)	0.51418 (10)	0.0192 (3)	
H7	−0.263572	0.393132	0.515024	0.023*	
C8	−0.21990 (13)	0.18053 (17)	0.55720 (10)	0.0191 (3)	
H8	−0.282715	0.156611	0.593911	0.023*	
C9	−0.31184 (15)	−0.1728 (2)	0.28848 (11)	0.0268 (4)	
H9A	−0.325502	−0.242347	0.241949	0.040*	
H9B	−0.377717	−0.101487	0.288064	0.040*	
H9C	−0.308875	−0.225503	0.342481	0.040*	
C10	−0.19552 (14)	−0.09537 (16)	0.27774 (10)	0.0195 (3)	
C11	−0.12922 (14)	−0.06840 (18)	0.20783 (10)	0.0206 (3)	
H11	−0.149648	−0.094806	0.150952	0.025*	
C12	−0.02518 (13)	0.00603 (17)	0.23726 (9)	0.0189 (3)	
C13	0.07636 (15)	0.0650 (2)	0.18861 (11)	0.0272 (4)	
H13A	0.101087	−0.008066	0.147390	0.041*	
H13B	0.144532	0.087654	0.227618	0.041*	
H13C	0.050240	0.154119	0.158824	0.041*	
C14	0.30155 (13)	0.17584 (17)	0.44170 (10)	0.0207 (3)	
C15	0.43466 (15)	0.2066 (2)	0.45627 (13)	0.0363 (4)	
H15A	0.473384	0.211363	0.401669	0.054*	
H15B	0.471550	0.128286	0.490694	0.054*	
H15C	0.445230	0.300123	0.485868	0.054*	
N1	0.05042 (11)	0.32623 (14)	0.37348 (8)	0.0158 (2)	
N2	−0.05658 (10)	0.16361 (14)	0.48562 (7)	0.0143 (2)	
N3	−0.12709 (11)	0.09133 (14)	0.53973 (8)	0.0157 (2)	
N4	−0.02724 (11)	0.02391 (14)	0.32099 (8)	0.0161 (2)	
N6	−0.13244 (11)	−0.03814 (14)	0.34435 (8)	0.0179 (3)	
O1	0.23062 (10)	0.19619 (12)	0.50069 (7)	0.0213 (2)	
O2	0.26366 (10)	0.12852 (12)	0.37041 (7)	0.0204 (2)	
Ni1	0.08662 (2)	0.11295 (2)	0.41577 (2)	0.01351 (7)	
H6	−0.1496 (16)	−0.046 (2)	0.3967 (12)	0.016*	

### Bis[µ-3-(pyridin-2-yl)pyrazolato]bis[acetato(3,5-dimethyl-1H-pyrazole)nickel(II)] Atomic displacement parameters (Å^2^)

**Table:**

	*U*^11^	*U*^22^	*U*^33^	*U*^12^	*U*^13^	*U*^23^
C1	0.0153 (7)	0.0217 (8)	0.0195 (7)	−0.0015 (5)	0.0013 (6)	0.0017 (6)
C2	0.0190 (7)	0.0229 (8)	0.0218 (8)	−0.0039 (6)	0.0009 (6)	0.0061 (6)
C3	0.0223 (8)	0.0164 (7)	0.0249 (8)	0.0001 (6)	−0.0022 (6)	0.0037 (6)
C4	0.0164 (7)	0.0188 (7)	0.0198 (7)	0.0007 (5)	−0.0014 (6)	−0.0008 (6)
C5	0.0139 (6)	0.0180 (7)	0.0136 (6)	−0.0012 (5)	−0.0024 (5)	−0.0015 (5)
C6	0.0146 (6)	0.0171 (7)	0.0143 (6)	−0.0001 (5)	−0.0008 (5)	−0.0009 (5)
C7	0.0171 (7)	0.0205 (8)	0.0200 (7)	0.0040 (6)	0.0022 (6)	0.0005 (6)
C8	0.0152 (7)	0.0234 (8)	0.0189 (7)	0.0029 (6)	0.0037 (5)	0.0013 (6)
C9	0.0225 (8)	0.0303 (9)	0.0271 (8)	−0.0091 (7)	−0.0076 (6)	0.0063 (7)
C10	0.0183 (7)	0.0166 (7)	0.0229 (8)	−0.0001 (5)	−0.0054 (6)	0.0023 (6)
C11	0.0212 (7)	0.0217 (7)	0.0183 (7)	0.0017 (6)	−0.0044 (6)	−0.0010 (6)
C12	0.0180 (7)	0.0200 (7)	0.0186 (7)	0.0032 (6)	−0.0006 (6)	0.0015 (6)
C13	0.0226 (8)	0.0396 (10)	0.0197 (8)	−0.0009 (7)	0.0034 (6)	−0.0011 (7)
C14	0.0144 (7)	0.0201 (7)	0.0275 (8)	−0.0013 (5)	−0.0002 (6)	0.0065 (6)
C15	0.0161 (8)	0.0456 (11)	0.0467 (11)	−0.0063 (7)	−0.0028 (7)	0.0064 (9)
N1	0.0134 (6)	0.0186 (6)	0.0154 (6)	−0.0008 (5)	−0.0005 (4)	0.0009 (5)
N2	0.0126 (5)	0.0166 (6)	0.0136 (6)	−0.0005 (5)	0.0013 (4)	0.0009 (5)
N3	0.0129 (6)	0.0196 (6)	0.0147 (6)	0.0006 (5)	0.0023 (4)	0.0016 (5)
N4	0.0134 (6)	0.0165 (6)	0.0183 (6)	0.0001 (4)	0.0011 (5)	0.0012 (5)
N6	0.0162 (6)	0.0204 (6)	0.0172 (6)	−0.0022 (5)	−0.0003 (5)	0.0016 (5)
O1	0.0176 (5)	0.0256 (6)	0.0205 (5)	−0.0023 (4)	−0.0009 (4)	0.0005 (4)
O2	0.0164 (5)	0.0240 (6)	0.0210 (6)	0.0005 (4)	0.0043 (4)	0.0027 (4)
Ni1	0.01067 (11)	0.01613 (12)	0.01377 (11)	−0.00014 (6)	0.00125 (7)	0.00074 (6)

### Bis[µ-3-(pyridin-2-yl)pyrazolato]bis[acetato(3,5-dimethyl-1H-pyrazole)nickel(II)] Geometric parameters (Å, º)

**Table:**

C1—N1	1.3393 (19)	C11—C12	1.405 (2)
C1—C2	1.382 (2)	C11—H11	0.9500
C1—H1	0.9500	C12—N4	1.3348 (19)
C2—C3	1.388 (2)	C12—C13	1.490 (2)
C2—H2	0.9500	C13—H13A	0.9800
C3—C4	1.387 (2)	C13—H13B	0.9800
C3—H3	0.9500	C13—H13C	0.9800
C4—C5	1.390 (2)	C14—O1	1.2576 (19)
C4—H4	0.9500	C14—O2	1.2641 (19)
C5—N1	1.3547 (19)	C14—C15	1.514 (2)
C5—C6	1.460 (2)	C14—Ni1	2.4738 (15)
C6—N2	1.3488 (19)	C15—H15A	0.9800
C6—C7	1.395 (2)	C15—H15B	0.9800
C7—C8	1.384 (2)	C15—H15C	0.9800
C7—H7	0.9500	N1—Ni1	2.0964 (13)
C8—N3	1.3514 (19)	N2—N3	1.3541 (17)
C8—H8	0.9500	N2—Ni1	2.0245 (12)
C9—C10	1.489 (2)	N3—Ni1^i^	2.0409 (13)
C9—H9A	0.9800	N4—N6	1.3626 (17)
C9—H9B	0.9800	N4—Ni1	2.0884 (12)
C9—H9C	0.9800	N6—H6	0.859 (18)
C10—N6	1.3479 (19)	O1—Ni1	2.1848 (11)
C10—C11	1.374 (2)	O2—Ni1	2.1232 (11)
			
N1—C1—C2	122.95 (15)	O1—C14—Ni1	61.93 (8)
N1—C1—H1	118.5	O2—C14—Ni1	59.12 (8)
C2—C1—H1	118.5	C15—C14—Ni1	177.13 (13)
C1—C2—C3	118.39 (14)	C14—C15—H15A	109.5
C1—C2—H2	120.8	C14—C15—H15B	109.5
C3—C2—H2	120.8	H15A—C15—H15B	109.5
C4—C3—C2	119.50 (14)	C14—C15—H15C	109.5
C4—C3—H3	120.2	H15A—C15—H15C	109.5
C2—C3—H3	120.2	H15B—C15—H15C	109.5
C3—C4—C5	118.75 (14)	C1—N1—C5	118.57 (13)
C3—C4—H4	120.6	C1—N1—Ni1	127.31 (10)
C5—C4—H4	120.6	C5—N1—Ni1	114.10 (10)
N1—C5—C4	121.80 (14)	C6—N2—N3	108.63 (12)
N1—C5—C6	114.08 (13)	C6—N2—Ni1	114.95 (10)
C4—C5—C6	124.12 (14)	N3—N2—Ni1	135.91 (10)
N2—C6—C7	109.55 (13)	C8—N3—N2	107.36 (12)
N2—C6—C5	117.19 (13)	C8—N3—Ni1^i^	129.94 (10)
C7—C6—C5	133.25 (14)	N2—N3—Ni1^i^	122.68 (9)
C8—C7—C6	103.89 (13)	C12—N4—N6	105.39 (12)
C8—C7—H7	128.1	C12—N4—Ni1	136.57 (11)
C6—C7—H7	128.1	N6—N4—Ni1	118.01 (9)
N3—C8—C7	110.57 (13)	C10—N6—N4	112.04 (13)
N3—C8—H8	124.7	C10—N6—H6	126.3 (12)
C7—C8—H8	124.7	N4—N6—H6	121.3 (12)
C10—C9—H9A	109.5	C14—O1—Ni1	87.55 (9)
C10—C9—H9B	109.5	C14—O2—Ni1	90.15 (9)
H9A—C9—H9B	109.5	N2—Ni1—N3^i^	100.88 (5)
C10—C9—H9C	109.5	N2—Ni1—N4	90.81 (5)
H9A—C9—H9C	109.5	N3^i^—Ni1—N4	90.53 (5)
H9B—C9—H9C	109.5	N2—Ni1—N1	79.39 (5)
N6—C10—C11	106.28 (14)	N3^i^—Ni1—N1	177.68 (5)
N6—C10—C9	121.53 (15)	N4—Ni1—N1	91.77 (5)
C11—C10—C9	132.18 (15)	N2—Ni1—O2	157.80 (5)
C10—C11—C12	106.26 (13)	N3^i^—Ni1—O2	89.05 (5)
C10—C11—H11	126.9	N4—Ni1—O2	109.07 (5)
C12—C11—H11	126.9	N1—Ni1—O2	89.92 (4)
N4—C12—C11	110.03 (14)	N2—Ni1—O1	99.02 (5)
N4—C12—C13	120.64 (14)	N3^i^—Ni1—O1	87.78 (5)
C11—C12—C13	129.31 (14)	N4—Ni1—O1	170.18 (5)
C12—C13—H13A	109.5	N1—Ni1—O1	89.91 (4)
C12—C13—H13B	109.5	O2—Ni1—O1	61.24 (4)
H13A—C13—H13B	109.5	N2—Ni1—C14	129.01 (5)
C12—C13—H13C	109.5	N3^i^—Ni1—C14	87.58 (5)
H13A—C13—H13C	109.5	N4—Ni1—C14	139.74 (5)
H13B—C13—H13C	109.5	N1—Ni1—C14	90.48 (5)
O1—C14—O2	121.02 (14)	O2—Ni1—C14	30.73 (5)
O1—C14—C15	119.71 (15)	O1—Ni1—C14	30.52 (5)
O2—C14—C15	119.27 (15)		
			
N1—C1—C2—C3	1.7 (2)	C7—C6—N2—N3	0.44 (16)
C1—C2—C3—C4	−2.0 (2)	C5—C6—N2—N3	−178.96 (12)
C2—C3—C4—C5	0.6 (2)	C7—C6—N2—Ni1	173.56 (10)
C3—C4—C5—N1	1.2 (2)	C5—C6—N2—Ni1	−5.84 (16)
C3—C4—C5—C6	−178.64 (14)	C7—C8—N3—N2	0.27 (17)
N1—C5—C6—N2	5.98 (19)	C7—C8—N3—Ni1^i^	178.58 (10)
C4—C5—C6—N2	−174.14 (13)	C6—N2—N3—C8	−0.43 (15)
N1—C5—C6—C7	−173.25 (15)	Ni1—N2—N3—C8	−171.45 (11)
C4—C5—C6—C7	6.6 (3)	C6—N2—N3—Ni1^i^	−178.89 (9)
N2—C6—C7—C8	−0.27 (17)	Ni1—N2—N3—Ni1^i^	10.09 (18)
C5—C6—C7—C8	179.01 (15)	C11—C12—N4—N6	−0.36 (17)
C6—C7—C8—N3	0.00 (17)	C13—C12—N4—N6	178.19 (14)
N6—C10—C11—C12	0.30 (17)	C11—C12—N4—Ni1	177.84 (11)
C9—C10—C11—C12	−178.23 (17)	C13—C12—N4—Ni1	−3.6 (2)
C10—C11—C12—N4	0.04 (18)	C11—C10—N6—N4	−0.55 (17)
C10—C11—C12—C13	−178.35 (16)	C9—C10—N6—N4	178.17 (14)
C2—C1—N1—C5	0.1 (2)	C12—N4—N6—C10	0.57 (16)
C2—C1—N1—Ni1	−178.23 (11)	Ni1—N4—N6—C10	−178.03 (10)
C4—C5—N1—C1	−1.6 (2)	O2—C14—O1—Ni1	1.96 (15)
C6—C5—N1—C1	178.30 (13)	C15—C14—O1—Ni1	−177.31 (14)
C4—C5—N1—Ni1	176.96 (11)	O1—C14—O2—Ni1	−2.01 (15)
C6—C5—N1—Ni1	−3.15 (15)	C15—C14—O2—Ni1	177.26 (14)

Symmetry code: (i) −*x*, −*y*, −*z*+1.

### Bis[µ-3-(pyridin-2-yl)pyrazolato]bis[acetato(3,5-dimethyl-1H-pyrazole)nickel(II)] Hydrogen-bond geometry (Å, º)

**Table:**

*D*—H···*A*	*D*—H	H···*A*	*D*···*A*	*D*—H···*A*
N6—H6···N2	0.859 (18)	2.571 (18)	2.9931 (18)	111.4 (14)
N6—H6···N3	0.859 (18)	2.591 (19)	3.3065 (18)	141.4 (16)
N6—H6···O1^i^	0.859 (18)	2.332 (19)	3.0800 (17)	145.6 (16)
C1—H1···O2	0.95	2.61	3.1281 (19)	115
C2—H2···O2^ii^	0.95	2.37	3.2692 (19)	158
C4—H4···O1^iii^	0.95	2.62	3.4696 (19)	149
C9—H9*C*···O1^i^	0.98	2.61	3.426 (2)	141
C13—H13*B*···O2	0.98	2.60	3.530 (2)	159

Symmetry codes: (i) −*x*, −*y*, −*z*+1; (ii) −*x*+1/2, *y*+1/2, −*z*+1/2; (iii) −*x*, −*y*+1, −*z*+1.
